# Editorial: Tissue engineering and regenerative medicine: advances, controversies, and future directions

**DOI:** 10.3389/fbioe.2025.1568490

**Published:** 2025-03-14

**Authors:** Stanislav Žiaran, Ľuboš Danišovič, Niels Hammer

**Affiliations:** ^1^ National Institute of Rheumatic Diseases, Piestany, Slovakia; ^2^ Department of Urology, Faculty of Medicine, Comenius University in Bratislava, Bratislava, Slovakia; ^3^ Faculty of Medicine, Institute of Medical Biology, Genetics and Clinical Genetics, Comenius University in Bratislava, Bratislava, Slovakia; ^4^ Division of Macroscopic and Clinical Anatomy, Gottfried Schatz Research Center, Medical University of Graz, Graz, Austria; ^5^ Department of Orthopedic and Trauma Surgery, University of Leipzig, Leipzig, Germany; ^6^ Division of Biomechatronics, Fraunhofer Institute for Forming Tools, Dresden, Germany

**Keywords:** bioprinting, biomaterials, regenerative medicine, stem cells, tissue engineering

## Advances in tissue engineering-based regenerative medicine - a vision for the future

Tissue engineering and regenerative medicine have undergone significant advancements over the last years, involving scientists, clinicians, and industrial partners alike. At the intersection of engineering, biology and medical science, interdisciplinary approaches will offer new therapeutic strategies from debilitating conditions to millions of patients suffering (Sahakyants and Vacanti, 2020). Three-dimensional bioprinting has been one of the most striking developments in tissue engineering over the last years. 4D and 5D bioprinting will soon allow for the precise fabrication of more complex tissue structure (Abolhassani et al., 2025). Such technology enabled creating of organoids, simple organs and cutting-edge tissue design, which offers new pathways in personalized medicine. Furthermore, advances in biomaterials design, including hydrogels or bioactive scaffolds, help mimic the extracellular matrix in a dynamic environment, facilitating and expediting tissue regeneration and ingrowth (Choi et al., 2024). Stem cell technology introducing pluripotent stem cells, providing ethically viable and patient-own cells in regenerative medicine. Advancements in acellularization and recolonizing tissues, structures and even organs comprising vasculature gain increasing attention. Using acellular scaffolds helps preserve the intrinsic tissue structure and main components of the formed backbone of the matrix (Verboket et al., 2024). When combined with patient-derived cell lines, such scaffolds offer promising platforms for tissue regeneration due to their low antigenicity. Bioreactors may help provide a highly controlled environment to facilitate the *ex vivo* growth of tissue-engineered scaffolds, which may soon become suitable at an industrial scale for clinical application.

Despite such developments, controversy and challenges persist. In addition to ethical constrains related to tissue harvesting, accessibility and affordability may form bottlenecks in prospective use. Scalability and reproducibility of tissue engineered scaffolds form further challenges. Lacking clinical trials and long-term experience will all become hurdles to make tissue engineered scaffolds become safe and robust solutions for the patients. Challenges lie even earlier in tissue engineering, as morpho-mechanical properties–both the existing ones and the desired ones for tissue engineered scaffolds–remain largely unknown. Standardized protocols at all steps of tissue engineering are needed to address this aspect.

## Sample size and the mechanical setup, considering tissue structure, hydration, anisotropy and heterogeneity

Tissue mechanical properties are strictly linked to their morphological features, on a scale ranging from macro to nano (Lozano et al., 2019). It is vital to understand how biological materials respond to both intrinsic and external forces to advance applications in tissue engineering. Using the combined knowledge of the relationship between tissue structure, composition, and load-deformation properties will help design tailored models, tissue scaffolds, and treatments for future clinical needs (Kuniakova et al., 2024).

Determining an appropriate sample size is crucial in biomechanical research, as it directly impacts reliability and reproducibility. Small samples reduce stability, while excessive sampling is impractical, particularly for human tissues (Hammer et al., 2023). Sample size depends on error tolerance, statistical methods, and morpho-mechanical properties, with high-precision studies often requiring large samples. Standardized methodologies are essential to account for post-mortem change and tissue heterogeneity, ensuring efficient resource use and reliable results for surgical and tissue-engineering advancements. Tissue anisotropy, heterogeneity, and hydration add complexity to biomechanical testing (Zwirner et al., 2020b). Collagen alignment, fiber density, and extracellular matrix composition influence properties like tensile strength and elastic modulus, while layered tissues enhance load resistance. Hydration also plays a critical role, as water content significantly affects deformation behavior (Lozano et al., 2019). These factors highlight the importance of controlled testing conditions.

Mechanical setups significantly affect testing accuracy, with sample geometry and clamping conditions playing a key role (Zwirner et al., 2020b). Tapered samples improve stress distribution, while advanced clamping technologies, such as resin-enforced and 3D-printed grips, reduce slippage and distortion, enhancing reproducibility (Scholze et al., 2018; Horvath et al., 2024). Load rates influence tissue stiffness and extensibility, reflecting viscoelastic behavior (Zwirner et al., 2023). Measurement innovations like digital image correlation provide accurate, non-intrusive data, further improving biomechanical modeling for surgical and scaffold applications (Zwirner et al., 2020a).

## Future directions

The future of tissue engineering and regenerative medicine lies in converging and leveraging emerging technologies. Artificial intelligence, machine learning, and automation are expected to accelerate progress by optimizing biomaterial design, predicting patient-specific outcomes, and refining bioprinting techniques. Gene-editing tools may unlock new treatment opportunities for genetic disorders, thereby enhancing tissue functionality.

To achieve these goals, effective interdisciplinary collaboration will be essential to overcome the limitations currently faced. Partnerships among engineers, biologists, scientists, and clinicians will play a pivotal role in ensuring that technological advancements are translated into safe, effective, and accessible therapies. Emphasizing collaboration, standardization, and alignment with policymakers could significantly expedite the transition to human trials and clinical practice.

While tissue engineering and regenerative medicine present significant challenges, they also offer vast opportunities. The full potential of this transformative field can only be realized through collaboration, as highlighted by the contributions featured Research Topic entitled “Tissue Engineering and Regenerative Medicine: Advances, Controversies, and Future Directions”.

Our Research Topic includes five articles addressing some of the most emerging questions: The review and research paper by Lin et al. assessed testing parameters for uniaxial testing of human soft tissues, as no standards exist to date for biological materials. They found broad variability in sample shape and clamping conditions, with subsequent numerical simulations suggesting that geometry impacts stress distribution, indicating the need for standardized reporting of the testing environment. Jiang et al. in their review explore the use of gene-modified induced mesenchymal stem cells (iMSCs) and enhanced MSC-derived exosomes as a potential therapeutic approach for the treatment of osteoarthritis and cartilage repair. Ansari et al. review different combinations of hydrogels, biomaterials, combined with advanced strategies such as drug delivery and mechano-signaling for cartilage repair, highlighting their potential to overcome the limitations of traditional osteoarthritis therapies. The study of Josino and Stimamiglio demonstrates that bioactive decellularized extracellular matrix-based hydrogel do support human adipose-derived stem cell maintenance and fibrocartilage phenotype. Jovic et al. found that isolated fibronectin-adherent chondroprogenitor cells did not offer significant benefits, highlighting the need for improved isolation methods and markers in future.
